# Visual routines are associated with specific graph interpretations

**DOI:** 10.1186/s41235-017-0059-2

**Published:** 2017-03-20

**Authors:** Audrey L. Michal, Steven L. Franconeri

**Affiliations:** Department of Psychology, Northwestern University, 2029 Sheridan Road, Evanston, IL 60208 USA

**Keywords:** Visual routines, Graph comprehension, Relations, Embodied cognition, STEM learning

## Abstract

We argue that people compare values in graphs with a *visual routine* – attending to data values in an ordered pattern over time. Do these visual routines exist to manage capacity limitations in how many values can be encoded at once, or do they actually affect the relations that are extracted? We measured eye movements while people judged configurations of a two-bar graph based on size only (“[short tall] or [tall short]?”) and contrast only (“[light dark] or [dark light]?”). Participants exhibited visual routines in which they systematically attended to a specific feature (or “anchor point”) in the graph; in the size task, most participants inspected the taller bar first, and in the contrast task, most participants attended to the darker bar first. Participants then judged configurations that varied in both size and contrast (e.g., [short-light tall-dark]); however, only one dimension was task-relevant (varied between subjects). During this orthogonal task, participants overwhelmingly relied on the same anchor point used in the single-dimension version, but only for the task-relevant dimension (e.g., taller bar for the size-relevant task). These results suggest that visual routines are associated with specific graph interpretations. Responses were also faster when task-relevant and task-irrelevant anchor points appeared on the same object (congruent) than on different objects (incongruent). This interference from the task-irrelevant dimension suggests that top-down control may be necessary to extract relevant relations from graphs. The effect of visual routines on graph comprehension has implications for both science, technology, engineering, and mathematics pedagogy and graph design.

**Electronic supplementary material:**

The online version of this article (doi:10.1186/s41235-017-0059-2) contains supplementary material, which is available to authorized users.

## Significance

Graphs are often considered intuitive because they display data visuospatially; yet, students still struggle. Because data comparisons are critical for many science, technology, engineering, and mathematics (STEM) tasks, we must learn the underlying reasons why graph comprehension can fail and how it might be improved. Here we explore a core visual mechanism for extracting relations from values in graphs. We argue that when people extract some types of relationships, as simple as, “Is *X* larger than *Y*?,” they must use a “visual routine” to extract it. Because data points can be compared in multiple ways (e.g., based on size, contrast, or spatial location), part of the difficulty of between-value comparisons may be that users must focus this routine on relevant comparisons. Here we show that, when there are multiple potential relations to extract from an otherwise extremely simple dataset, visual routines are associated with specific relational comparisons. If the location and order of attention through a graph are associated with the relations that are extracted, then there may be a role for teaching the “right” ordering to students.

## Background

Graphical literacy – the ability to read, construct, and interpret visual displays of information – is a critical skill for all citizens, but particularly for those in STEM fields. Everyday STEM tasks require people to judge whether information in a graph supports a claim, identify the cause of a problem on the basis of anomalous data, and extrapolate trends to predict future performance. Although graphs are often perceived as intuitive because they group relevant information together in a visuospatial format (e.g., Larkin & Simon, 1987), graph interpretation requires analysis of the parts of a graph over time, more akin to slowly reading a paragraph than to glancing at a picture (e.g., Carpenter & Shah, 1998). Because of visual capacity limitations, the relations that people extract from graphs may be tightly constrained by the order in which they attend to graphical elements.

Specifically, we propose that extracting a between-value graph comparison (e.g., “Are there more *X* than *Y*?”) elicits a serial operation that we refer to as a *visual routine* (Cavanagh, 2004; Ullman, 1984) in which attention shifts to at least one of the objects in the relation to guide the comparison process. For instance, when judging whether a two-bar graph depicts a specific relation configuration (i.e., [short tall] or [tall short]?), most people either systematically attend to the left bar first or the taller bar first (Michal, Parrot & Franconeri: Three modes for seeing relations between objects, in preparation). The requirement of using a visual routine to extract such relations is not unique to graphs – people also judge color configurations of objects (e.g., “green circle left of red circle?”) by shifting attention to one of the colored objects (Franconeri, Scimeca, Roth, Helseth, & Kahn, 2012; Holcombe, Linares, & Vaziri-Pashkam, 2011; Yuan, Uttal, & Franconeri, 2016).

These visual routines may be needed because of exceptionally tight capacity limits on these types of visual relation extraction (Logan, 1994, 1995; Wolfe, 1998), with one proposal requiring strictly serial processing of objects within a relation (Franconeri et al., 2012). In order to judge a particular spatial relation between two objects (e.g., “Is the larger object on the left?”), one object has to be designated as the *target* (e.g., the larger object) and one object has to be designated as the *referent* (e.g., the smaller object). Although the individual features of multiple objects may initially be available at a global, scene-statistic level, such as knowing there are two sizes, two contrast values, and two locations in a display (e.g., Alvarez, 2011), the visual system must have a mechanism for assigning features belonging to the target object and referent object, and one likely mechanism is strict spatial isolation of attention across temporal intervals (Franconeri et al., 2012, Treisman & Gelade, 1980; but see Hummel & Biederman, 1992). Thus, when judging whether the left of two objects is the larger object, a visual routine would isolate the two features “larger” and “left” to a single object, making the location of the larger object explicit.

**Fig. 1:**
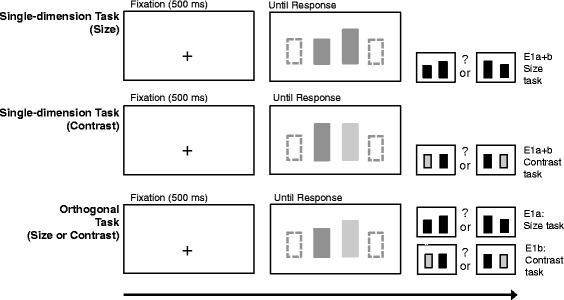
Example task sequences for the size single-dimension task, contrast single-dimension task, and orthogonal task. Bars could appear in any of the four outlined positions but were always directly adjacent to one another. For the orthogonal task, half of the participants responded to the size relation only and half responded to the contrast relation only. Bar stimuli and positions are not drawn to scale

Past evidence shows that visual routines occur during graph comprehension (e.g., Michal, Uttal, Shah, & Franconeri, 2016), but converging evidence is needed to show that these routines are instrumental for extracting specific relations. For example, when judging the size configuration of the bars in the top graph of Fig. 1, we claim that looking toward the taller bar first allows the viewer to extract the relation “the taller bar is on the right,” rather than “the shorter bar is on the left.” That claim requires evidence that the type of relation judged affects the way that attention shifts (as reflected in eye movements).

In support of this idea, we recently found that interpretations of magnitude relations depended on which item people attended to first (Michal et al., 2016). Participants were asked to verify whether a two-bar graph matched a statement such as, “Are there more blueberries than oranges?,” and visual routines that mimicked the linguistic order within the question led to faster responses. By attending first to the relational target (e.g., the blueberry bar), people were more likely to interpret the graph in a way that was aligned with the question (i.e., they interpreted the graph as “more blueberries than oranges” rather than “fewer oranges than blueberries”), which led to quicker responses.

Because Michal et al. (2016) asked participants to verify a specific framing of a visual relation (e.g., blue bar larger than orange bar?), participants may have been primed to attend to the graph using a sentence-order visual routine. We must therefore additionally show that people naturally interpret graphs in a sentence-like way even when there is no verbal statement to compare the graph to, and when they are free to attend to the graphed values in any temporal order they choose.

One way to test whether visual routines are associated with specific relational comparisons is to ask people to judge relations for which there are multiple ways to compare the two objects – and therefore multiple potential routines. For example, although the bottom graph in Fig. 1 (under the words *until response*) has only two bars, one could compare the bars’ locations, sizes, or contrast values. Each comparison can also be described on the basis of different dimensions, such as “the taller bar is on the right” or “the darker bar is on the left.” Finally, each comparison can be framed differently; for instance, judging that “the taller bar is on the right” is distinct from “the shorter bar is on the left” (e.g., Clark & Chase, 1972). Thus, when there are several possible ways of comparing data points in a graph, it is critical to be able to make relevant comparisons. For instance, if a person wanted to verify whether the right bar was larger than the left bar in the bottom graph of Fig. 1, they would need to compare the sizes of the bars and not the contrasts or spatial locations.

We asked participants to judge configurations of a two-bar graph on the basis of size (i.e., “[short tall] or [tall short]?”) and contrast values (i.e., “[light dark] or [dark light]?”). By measuring which bar participants attended to first over a range of display types, we could infer the features or “anchor points” (Couclelis, Golledge, Gale, & Tobler, 1987) that guided their comparisons. If people are systematic about the feature that they attend to first, then it is likely that their interpretation of the graph is driven by that guiding feature. For example, a typical observer judging the top graph in Fig. 1 might use the taller bar as an anchor point when comparing the sizes of the bars, whereas she might use the darker bar as an anchor point when comparing the contrasts (middle graph of Fig. 1). But do these visual routines happen simply because of the relative bottom-up salience of those feature values, or are the saccades guided by the relational decision in a top-down manner? The example in the lowest graph of Fig. 1 pits the two strategies of attending to the taller or darker bar first against each other because the graph varies in both size and contrast, but only one dimension is task-relevant. Thus, if visual routines are associated with specific graph interpretations, then the routine used to compare two data points should be based on whichever dimension is currently relevant for the comparison. If a viewer prefers dark and tall, then in this display they should attend left when judging contrast versus right when judging size, even in otherwise identical displays.

By measuring the target object of first saccades over a range of configurations, we first identified anchor points for each participant while they judged graph configurations on the basis of size (with task-irrelevant contrast kept constant) and contrast (with task-irrelevant size kept constant). After completing these single-dimension tasks, participants completed an orthogonal task in which graphs varied in both size and contrast configurations. Half of the participants judged size configurations while task-irrelevant contrast values varied independently (Experiment 1a), and the other half judged contrast configurations while task-irrelevant size values varied independently (Experiment 1b). If visual routines are guided by the task-relevant dimension (size or contrast) during graph comprehension, then people should show the same anchor point biases for the orthogonal task as for the corresponding single-dimension task. Furthermore, if visual routines are implemented in a task-relevant way, then the order of this visual routine might affect how people interpret relations in graphs; if so, then exploring the “right” order for a given problem could have pedagogical implications.

## Methods

### Experiments 1a and 1b

#### Participants

We collected data from 36 healthy participants with normal or corrected-to-normal vision from the Northwestern University community. All participants provided informed consent to participate in the study, which was approved by the Northwestern University Institutional Review Board. Participants were either compensated $10 or received course credit for participating.

#### Apparatus

All stimuli were created using MATLAB software (MathWorks, Natick, MA, USA) and displayed using Experiment Builder (SR Research, Ottawa, ON, Canada) on a Mac mini (Apple, Cupertino, CA, USA) with an 18-inch ViewSonic CRT monitor (1024 × 768 pixels, 85 Hz; ViewSonic, Walnut, CA, USA). Eye movements were recorded by an EyeLink 1000 Tower Mount eye tracker (1000-Hz sampling rate; SR Research). Participants were seated approximately 81 cm from the display.

#### Procedure and stimuli

All participants performed three bar graph judgment tasks: a size single-dimension task (Are the bars arranged [short tall] or [tall short]?), a contrast single-dimension task (Are the bars arranged [light dark] or [dark light]?), and an orthogonal task (graphs varied in both size and contrast, but only one dimension was task-relevant). Nineteen participants performed the size-relevant version of the orthogonal task (Experiment 1a), and seventeen participants performed the contrast-relevant version of the orthogonal task (Experiment 1b). Participants completed both single-dimension tasks first (size and contrast, starting task counterbalanced across participants), followed by one randomly assigned orthogonal task. For all tasks, participants were instructed to fixate their eyes on a central fixation cross (positioned 12.6 degrees from the left and 9.4 degrees from the top of the screen) at the beginning of each trial, at which point a drift correction occurred. After 500 milliseconds, the stimuli appeared, and participants were free to move their eyes around the screen. The stimuli were displayed until participants made their response (Fig. 1).

For the size single-dimension task, participants viewed pairs of dark gray rectangular bars (luminance = 22 candelas [cd]/m^2^) presented on a light gray background (luminance = 55.6 cd/m^2^). The bars were 1.5 degrees wide and ranged in height from 1.3 degrees to 5.5 degrees, and the base of each bar was positioned 1.9 degrees below fixation. Each pair contained one shorter bar (14 possible heights ranging from 1.5 degrees to 3.9 degrees) and one taller bar (14 possible heights ranging from 1.8 degrees to 5.5 degrees), and the height ratio between the two bars in each pair was kept constant at 0.71. The bars were displayed in one of two configurations: [short tall] or [tall short]. Response keys depicted drawings of bar configurations (Fig. 1, top graph), and participants were instructed to press the key that matched the size configuration displayed. There were equal numbers of each configuration type, and the locations of the keys (left/right side of the keyboard) alternated across participants. To discourage participants from using absolute spatial location to locate the bar pairs, we randomly varied the bars’ locations across four possible regions such that a pair of bars occupied adjacent locations (left bars centered over positions 7.5 degrees and 2.5 degrees to the left of fixation; center bars centered over positions 2.5 degrees to the left and 2.5 degrees to the right of fixation; right bars centered over positions 2.5 degrees and 7.5 degrees to the right of fixation). However, we anticipated that the center two locations would be the most informative for distinguishing between eye movement preferences for the left versus right bar because the bars were distributed across the left and right visual fields only in these locations; thus, bars appeared in the center two positions twice as often as in the left and right positions, and only the central locations were included in the eye movement analysis.

In the contrast single-dimension task, participants viewed pairs of bars that varied in contrast from light gray (luminance = 88.8 cd/m^2^) to dark gray (luminance = 23.1 cd/m^2^) and were presented on a white background. The bars’ width and height were kept constant (1.5 degrees × 3.9 degrees); however, the contrast of each bar within a pair differed such that one bar was lighter (ranging in luminance from 35.6 cd/m^2^ to 88.8 cd/m^2^) and the other was darker (ranging in luminance from 23.1 cd/m^2^ to 78.5 cd/m^2^). The contrast ratios between the two bars were approximately 0.71 in red, green, blue (RGB) voltage-value space (note that this does not entail a 0.71 ratio after the RGB values are nonlinearly translated to luminance values). The bars were presented in one of two configurations: [light dark] or [dark light]. Participants were instructed to press the key that matched the contrast configuration of each bar pair (Fig. 1, middle graph). Key locations were counterbalanced across participants. As in the size task, we randomly varied the positions of the bar pairs, doubled the number of central position trials, and included only the central positions in the analysis.

In the orthogonal task, participants viewed bars that varied randomly in both size and contrast on a white background (Fig. 1, bottom graph). Within each pair, one bar was shorter and one taller, and one bar was lighter and one darker; thus, there were four possible configurations ([short-light tall-dark], [short-dark tall-light], [tall-light short-dark], and [tall-dark short-light]). The possible size and contrast values, ratio pairings, bar widths, and possible locations were identical to those in the single-dimension tasks. The instructions were also identical (judge the size or contrast configuration, depending on the relevant dimension), except that participants were told to ignore changes in the irrelevant dimension (i.e., ignore contrast variations in Experiment 1a, and ignore size variations in Experiment 1b).

Participants performed 1 block of practice trials for all 3 tasks, followed by 4 blocks of 28 trials for a total of 140 trials in the size and contrast tasks and 6 blocks of 28 trials for a total of 196 trials in the orthogonal task. The eye tracker camera was calibrated using a 9-point calibration procedure at the beginning of each task and in between each block of trials. To emphasize high accuracy, participants received auditory feedback and experienced a 6-second timeout for incorrect responses.

## Results

### Inclusion criteria

Saccades were defined by a velocity threshold of 30 degrees per second and by an acceleration threshold of 8000 degrees per second squared (Stampe, 1993). We excluded first saccades that were smaller than 0.5 degrees because this was the eye tracker accuracy provided by the manufacturer. We also excluded first saccades that were directed upward or downward such that the endpoint fell within a boundary defined by two diagonal lines, one starting at the highest possible point of the inner edge of the left bar (3.6 degrees above fixation) and crossing through the center to below the inner edge of the right bar (1.9 degrees below fixation), and the other starting above the highest possible point of the inner edge of the right bar (3.6 degrees above fixation) and crossing through the center to below the inner edge of the left bar (1.9 degrees below fixation). Finally, we excluded any saccades that started less than 100 milliseconds after stimulus onset (Fischer & Ramsperger, 1984). Of all first saccades, 20.1% were excluded in the size single-dimension task, 11.0% were excluded in the contrast single-dimension task, 18.8% were excluded in the size version of the orthogonal task, and 9.1% were excluded in the contrast version of the orthogonal task. We further excluded data of participants who made saccades on fewer than five trials to centrally located bars for either of the single-dimension tasks. On the basis of this criterion, a total of 10 participants were excluded; thus, data of 26 participants were included in the final analyses (*n* = 14 in Experiment 1a and *n* = 12 in Experiment 1b).

### Saccade latency

Average saccade latency relative to stimulus onset was 271 milliseconds for the size single-dimension task and 259 milliseconds for the contrast single-dimension task, and saccade latency values were statistically similar between the two tasks (*p* > .25). Additionally, average saccade latency was 263 milliseconds for the size-relevant orthogonal task and 244 milliseconds for the contrast-relevant orthogonal task; again, saccade latency values were statistically similar between the two versions of the task (*p* > .25).

### Classification of anchor points

**Fig. 2:**
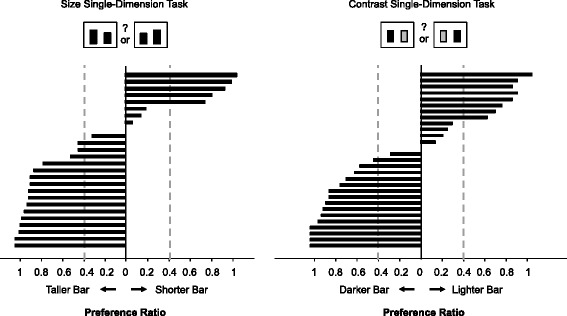
Anchor point eye movement analyses for the size (*left*) and contrast (*right*) single-dimension tasks. Data points represent the preference ratio ([number preferred −number nonpreferred]/[number preferred + number nonpreferred]) for each participant. Biases toward the taller bar and darker bar are plotted to the left, and biases toward the shorter and lighter bar are plotted toward the right. *Gray dashed lines* represent the threshold value (0.4) for having a bias toward a specific feature

We first determined each participant’s size anchor point (taller or shorter bar) and contrast anchor point (darker or lighter bar) from the single-dimension tasks. For each trial, we measured which bar participants looked at first relative to central fixation on the basis of size and contrast features of the bar, collapsing over the bar’s location. We categorized participants’ anchor points on the basis of the ratio of the number of saccades directed toward one feature over the over (preference ratio = [number of preferred saccades − number of nonpreferred saccades]/[number of preferred saccades + number of nonpreferred saccades]). Histograms of participants’ preference ratios for both the size and contrast single-dimension tasks are shown in Fig. 2. Biases toward one feature were defined as having a preference ratio value of at least 0.4 (equivalent to 70% of saccades directed toward one feature).

On the basis of this analysis, the majority of participants who had a clear anchor point in the size single-dimension task preferred the taller bar (16 of 21 participants), and the majority of participants who had a clear anchor point in the contrast single-dimension task preferred the darker bar (15 of 19 participants). We expected that most participants would show these preferences, owing to the relatively greater magnitude of the taller bars, which extended upward from just below the fixation point, and the relatively greater contrast of the darker bars, which appeared on a white background. However, not all subjects chose the taller and darker bar as their preferred anchor points; there were instances of each possible anchor point combination (tall-dark, tall-light, short-dark, and short-light; see Additional file 1 for visualizations of eye movements for four sample participants showing each of these preference combinations). Additionally, not all participants had a clear anchor point for each dimension: One participant had no feature preference for the size single-dimension task only; three participants had no feature preference for the contrast single-dimension task only; and four participants did not have a feature preference for either task. Details about subjective reports of feature preferences are included in Additional file 2.

### Eye movements

**Fig. 3:**
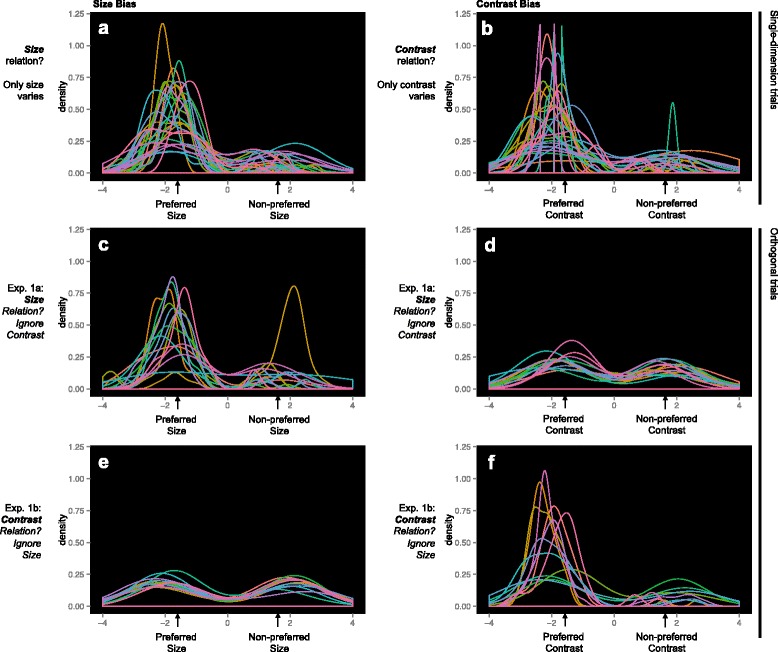
Probability density functions of size biases (**a**, **c**, **e**) and contrast biases (**b**, **d**, **f**) for the size single-dimension task (**a**), contrast single-dimension task (**b**), and orthogonal task (**c–f**). Plots depict length (in degrees of visual angle) of first saccades toward the preferred (negative *x*-axis) and non-preferred (positive *x*-axis) features relative to central fixation (0 degrees). *Vertical arrows* point to approximate locations of the inner edges of bar stimuli. Each *colored line* represents data of a single participant

We also measured participants’ feature biases during the orthogonal task by calculating preference ratios for each dimension (on the basis of preferred features used during the single-dimension tasks). If attentional shifts to a particular anchor point are associated with specific interpretations of spatial relations, then during the orthogonal task participants should show stronger preferences for anchor points defined by the task-relevant rather than the task-irrelevant single-dimension task. Figure 3 depicts density plots of all first saccades for all participants, with saccades toward the preferred feature plotted toward the left. In both single-dimension tasks, the peaks of the density plots are centered around the preferred feature, showing that anchor point biases were highly consistent across trials for most participants. Critically, the density plots for the orthogonal tasks illustrate that participants were generally biased to use whichever anchor point was idiosyncratically task-relevant for the orthogonal task (Fig. 3c–f). The saccade distributions based on size biases show a clear peak around the preferred size for almost every participant in Experiment 1a (size-relevant, Fig. 3c) but not in Experiment 1b (contrast-relevant, Fig. 3e). The reverse is true for saccade distributions based on contrast biases, with peaks occurring around the location of the preferred contrast for participants in Experiment 1b (Fig. 3f) but not in Experiment 1a (Fig. 3d).

**Fig. 4:**
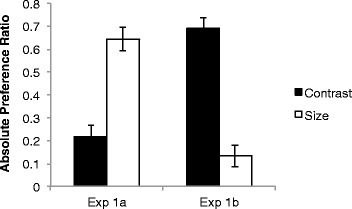
Absolute values of preference ratios based on contrast and size for the orthogonal task in Experiment 1a (size-relevant) and Experiment 1b (contrast-relevant). Error bars are based on within-subject SEM (Cousineau, 2005)

We verified the qualitative patterns illustrated in Fig. 3 quantitatively by testing whether preference ratios defined by the task-relevant dimension (based on anchor point preferences from the task-relevant single-dimension task) were more systematic than preference ratios defined by the task-irrelevant dimension (based on anchor point preferences from the task-irrelevant single-dimension task) during the orthogonal task. Specifically, we conducted a repeated-measures analysis of variance (ANOVA) on the absolute values of preference ratios with task (size-relevant, contrast-relevant) as a between-groups factor and preference ratio dimension (size, contrast) as a within-subject factor. There was a significant interaction between task and preference ratio dimension [*F*(1,24) = 50.24, *p* < .001]; post hoc tests revealed that absolute preference ratios in the size-relevant orthogonal task were significantly higher when they were defined by size anchor points than when defined by contrast anchor points [*t*(13) = 4.22, *p* < .01], whereas absolute preference ratios in the contrast-relevant orthogonal task were significantly higher when they were based on contrast anchor points than when based on size anchor points [*t*(11) = 6.02, *p* < .001] (Fig. 4). Thus, participants’ eye movements were guided primarily by anchor points from the task-relevant dimension during the orthogonal task.

### Behavioral results

Because attentional control is rarely perfect, it is possible that anchor points from the task-irrelevant dimension still influenced behavioral responses in the orthogonal task. For instance, a person who prefers tall and dark features may find it easier to judge a size configuration when the taller bar is also the darker bar rather than the lighter bar, even if the contrast dimension is task-irrelevant. Thus, we tested whether participants performed better on trials in which preferred features from both dimensions appeared on the same object versus different objects.

We further excluded eight participants from this analysis because they did not have clear anchor points for at least one of the single-dimension tasks, and we excluded one participant who used a different anchor point in the orthogonal task as the relevant single-dimension task (but still from the task-relevant dimension). On the basis of these inclusion criteria, the behavioral analyses included nine participants from Experiment 1a and eight participants from Experiment 1b. Congruent trials included displays in which a given participant’s size and contrast anchor points occurred in the same item, collapsed across left and right positions (e.g., congruent trials for a participant with a bias toward tall-dark items included trials with [dark-tall light-short] and [light-short dark-tall] configurations). In contrast, incongruent trials included displays in which a given participant’s anchor points appeared in different items (e.g., incongruent trials for a participant biased toward tall-dark items included trials with [dark-short light-tall] and [light-tall dark-short] configurations).

### Accuracy

Overall accuracy on the orthogonal task (collapsed across Experiments 1a and 1b) across all four stimulus locations was high, with only a 0.89% error rate. Further details about the congruency analysis for accuracy data are included in Additional file 2.

### Response times

Only trials for which responses were correct were included in the response time (RT) analyses. Additionally, we excluded any trials in which the RT was greater than 2 SD above the mean RT for each participant; in total, we excluded 6.3% of the dataset. To test for congruency effects of anchor points from both dimensions, we ran a repeated-measures ANOVA on RT data with experiment group (1a/1b) as a between-subjects factor and congruency (congruent/incongruent) as a within-subject factor. Across all four stimulus locations, there was a trend for a main effect of congruency, such that responses were faster congruent trials (*M* = 558 milliseconds) than in incongruent trials (*M* = 596 milliseconds) [*F*(1, 15) = 4.21, *p* = .06], and congruency effects were statistically similar between the two experimental groups (interaction *F* < 0.5). However, there was a significant main effect of congruency when we included only data from the two center locations [congruent *M* = 532, incongruent *M* = 581, *F*(1,15) = 14.34, *p* < .01; interaction *F* < 2]. We further established that congruency effects for the center two locations persisted throughout the experiment and were not driven by similar response mappings between the size and contrast single-dimension tasks (see Additional file 2 for details). Additionally, congruency effects were present as early as the first attentional shift for both versions of the orthogonal task and as early as the saccade initiation stage, but only for the size-relevant task (see Additional file 2 for details). Together, these results suggest that participants experienced behavioral interference from the task-irrelevant dimension; thus, the ability to extract relevant relations in graphs may require well-developed top-down control, particularly when graphs vary along multiple dimensions.

## Discussion

People tend to process graph relations using systematic visual routines, particularly when coordinating data with text (e.g., Michal et al., 2016). Here we show that people exhibited idiosyncratic but highly consistent feature preferences (“anchor points”) to guide visual routines when judging graph relations, even when there was no verbal component to the task. Most participants judged size configurations by attending to the taller bar first, whereas most participants judged contrast configurations by attending to the darker bar first. Importantly, these anchor points persisted in a task-dependent manner: When participants judged graphs that varied in both size and contrast (orthogonal task), preference ratios defined by the task-relevant dimension were significantly stronger than preference ratios defined by the task-irrelevant dimension. Thus, when a graph can be interpreted in multiple ways, people attend to a single dimension (e.g., size) in a specific order (e.g., taller bar first), which may generate a specific interpretation of the graph (e.g., “the taller bar is on the left”). These results are consistent with the proposal that visual routines do not merely facilitate graph comparisons by deploying attention to data points over time; rather, specific visual routines are associated with specific graph interpretations.

Although visual routines were implemented in a task-dependent manner, we cannot determine on the basis of our data whether multiple relations were processed in serial or parallel. However, we speculate that relational comparisons may be computed in serial for two reasons. First, serial processing is a parsimonious solution to the problem of binding separately processed features to single objects (Treisman & Gelade, 1980). If that solution were implemented during graph processing, then focused attention would be required to compute relations over those bound features (e.g., the user only explicitly knows that the left object is the taller one by shifting attention to a single object). Judging a spatial relation that is based on multiple dimensions would then require localization of multiple features that may or may not appear in the same location (e.g., “Where is the taller bar?” plus “Where is the darker bar?”); thus, we think it is unlikely that multiple relations could be judged in a single shift of attention. Second, in a change detection study, when we asked participants to detect changes to two relations instead of only one relation, performance was substantially impaired (Michal & Franconeri: Higher-order relational comparisons are limited to a single dimension at a time, in preparation). Although the present findings cannot confirm whether multiple relational comparisons are processed serially, our results are consistent with a serial mechanism.

Although eye movements were guided primarily by anchor points from the task-relevant dimension in the orthogonal task, variations in the task-irrelevant dimension did affect behavioral responses: Participants responded more quickly, on average, when their task-irrelevant anchor point appeared on the same object (congruent) than when it appeared on a different object (incongruent) as their task-relevant anchor point. Participants may have responded more slowly on incongruent trials if they were tempted to judge the task-irrelevant relation or momentarily forgot which dimension was task-relevant. Thus, despite the fact that visual routines were based on the task-relevant dimension, the task-irrelevant dimension competed for attention during the orthogonal task.

Given that most participants preferred the darker and taller objects, it is difficult to disentangle whether our RT findings reflect interference from the task-irrelevant relation or the relatively greater bottom-up salience of displays in which one object was both darker and taller. However, even if the interference effects were driven by low-level salience, it would still suggest that top-down control is necessary for extracting task-relevant comparisons. In other words, there would still be more competition for attention when salient features appeared on different objects than on the same object; thus, in either case, people need to be able to focus on the task-relevant feature to make a specific type of graph comparison. Importantly, this suggests that people may need to exert top-down control in order to focus on relevant comparisons in graphs.

Viewing patterns interact with cognitive performance in a range of tasks. For example, encouraging relevant eye movement patterns can prime successful problem solving (Grant & Spivey, 2003). In these studies, the claim was that an embodied signal from the eye movements themselves facilitated cognitive processing. In the present study, it is possible that people judged configurations in part by noting the direction of their own eye movements (Franconeri et al., 2012). For instance, when judging the size configuration of two bars, we posit that the size features are processed independently from their locations initially without linking which size appears at which location; however, the act of moving the eyes toward the taller bar would then allow one to infer the bar’s relative location (e.g., left) from the direction of the eye movement (e.g., leftward).

Overt eye movements would not be necessary to harness this embodied signal; the visual system could rely instead on the covert attentional shifts that typically accompany eye movements but are also made in the absence of eye movements. For instance, although we excluded data from participants who did not make a sufficient number of eye movements toward centrally presented bar stimuli, it is possible that these participants were still shifting attention covertly to individual bars when judging their relations. Our results are consistent with the idea that visual routines are embodied within the attentional shifts that typically—but do not always—accompany eye movements. Relations between objects are coded as the direction of motor movements that we would make to fixate on them.

If graph interpretations are driven by embodied visual routines, then there are several direct implications for how to best teach students to extract relevant information in graphs. First, students should focus on whichever comparison is currently task-relevant, which is a skill in itself that students can be taught (e.g., Richland, Morrison, & Holyoak, 2006). Second, as a general strategy for interpreting graphs in an open-ended way (i.e., they are not being asked about a specific directional relationship), students should choose one task-relevant anchor point to attend to first (e.g., the larger data point when comparing sizes) rather than attending to data points in an arbitrary order. By attending to graphs using a task-relevant visual routine and implementing that routine systematically, students may be able to improve their ability to focus on relevant graph comparisons.

## Conclusions

In conclusion, although individuals vary extensively in how they implement them, visual routines are strongly associated with specific graph interpretations. However, relations defined by other dimensions can interfere with the deployment of spatial attention. Thus, top-down control may be necessary to inhibit task-irrelevant comparisons; for instance, a person who can successfully ignore the contrast relation in the bottom graph of Fig. 1 may find it easier to judge the size configuration than a person who cannot inhibit the contrast relation. Further work is necessary to test whether selectively attending to relational structures is critical for both first-order (e.g., comparing one pair of bars) and higher-order comparisons in graphs (e.g., comparing two pairs of bars), and whether increased top-down control can improve conceptual reasoning about graphs.

## Additional files

Additional file 1: Additional information about subjective reports of anchor point preferences and anchor point congruency analyses. (DOCX 104 kb)

Additional file 2: Figure S1. Visualizations of first saccades for four representative participants for each single-dimension task and for the orthogonal task. For the orthogonal task, size was task-relevant for Participants 1 and 2, and contrast was task-relevant for Participants 3 and 4. Examples of incongruent trials (anchor points appearing on different bars) are shown for the orthogonal task (e.g., Participant 1 = tall/light bar; Participant 2 = short/dark bar; Participant 3 = tall/dark bar; Participant 4 = short/light bar). (PDF 265 kb)
