# Supplementary material for: Visual routines are associated with specific graph interpretations
Source: Cogn Res Princ Implic. 2017 Mar 20;2:20. doi: 10.1186/s41235-017-0059-2 (PMC5357663; doi:10.1186/s41235-017-0059-2)
Supplement: Supplementary file 1 — Additional information about subjective reports of anchor point preferences and anchor point congruency analyses. (DOCX 104 kb) [file 41235_2017_59_MOESM1_ESM.docx]

Supplementary Materials

*Method*

*Classification of eye movements.* We collected participant’s reported preferences for both the size and contrast *single-dimension* tasks because we wanted to determine the extent to which visual routines reflected explicit strategies. The subjective reports of preferred anchor points were consistent with our eye-movement-derived categorizations, with a few exceptions (one participant’s eye movements showed a tall bias but the subjective report stated no size preference; one participant’s eye movements showed a tall bias but the report stated a short bias; one participant’s eye movements showed a short bias but the report stated having a tall bias; one participant’s eye movements did not show a clear bias for either dimension but the report stated having a short bias and a light bias; one participant’s eye movements did not show a clear bias for either dimension but the report stated having a tall bias and a light bias; one participant’s eye movements showed a dark bias but the report stated no contrast preferences; one participant’s eye movements did not show a clear bias for either contrast feature but the report stated having a light bias; one participant’s eye movements did not show a clear bias for either contrast feature but the report stated having a dark bias). These results suggest that individuals may vary in the extent to which they are aware of using systematic attention strategies and what those strategies are.

Although most participants used the taller and darker bar as anchor points, as shown in Supplemental Figure 1, which depicts visualizations of all first saccades for four representative participants, there were instances of each possible anchor point combination (tall/dark, tall/light, short/dark and short/light).

----------------------------------------Supplemental Figure 1 Here----------------------------------

**Figure S1.** Visualizations of first saccades for four representative participants for each *single-dimension* task and for the *orthogonal* task. For the *orthogonal* task, size was task-relevant for Participants 1 & 2, and contrast was task-relevant for Participants 3 & 4. Examples of incongruent trials (anchor points appearing on different bars) are shown for the *orthogonal* task (e.g., Participant 1 = tall/light bar; Participant 2 = short/dark bar; Participant 3 = tall/dark bar; Participant 4 = short/light bar).

*Results*

*Accuracy.* We tested for congruency effects on accuracy data during the *orthogonal* task, where displays in which anchor points from each dimension appeared on the same object were defined as *congruent*, and displays in which anchor points from each dimension appeared on different objects were defined as *incongruent*. We ran a repeated measures ANOVA on error rate data with experiment group (1a/1b) as a between-subjects factor and congruency (congruent/incongruent) as a within-subjects factor. Error rates did not differ between the size-relevant (*M* = 0.76%) and contrast-relevant (*M* = 1.04%) versions of the task, nor did they differ between congruent (*M* = 0.93%) and incongruent (*M* = 0.88%) trials, and there was no interaction between the two groups, all *F*’s < 1. Similarly, when only trials in which the bars appeared in the two center locations were included, there were no significant effects, all *F*’s < 2.

*Response times.* In addition to analyzing congruency effects on RTs overall, we tested whether the congruency effect for the center locations persisted throughout the experiment. Specifically, we calculated mean RTs for congruent and incongruent trials separately for the first and second halves of the experiment and ran a repeated-measures ANOVA on RTs with experimental group (1a/1b; between-subjects), congruency (congruent or incongruent; within-subjects) and data subset (first or second half; within-subjects) as factors. There was a main effect of congruency, *F*(1,15) = 13.32, *p* < 0.01, but importantly, there was no interaction between congruency and data subset, *F* < 0.5, suggesting that congruency effects were stable across trials.

    We also tested the possibility that the congruency effect might be driven by similar response mappings between the *single-dimension* size and contrast tasks. For instance, if the left and right keys for a given participant displayed a [short tall] and [tall short] configuration respectively for the size *single-dimension* task and a [light dark] and [dark light] configuration respectively for the contrast *single-dimension* task, then that participant might learn to associate *short* with *light* and *tall* with *dark*. This response mapping association might then lead to an advantage for response-congruent displays (i.e., [short-light tall-dark] and [tall-dark short-light]) in the *orthogonal* task. However, because we counterbalanced the spatial arrangement of the response keys, response congruency did not perfectly correlate with anchor point congruency (e.g., there were some participants who preferred dark/tall features but their response keys associated light with tall). In contrast to the anchor point congruency analysis, RTs were statistically similar for response-congruent and response-incongruent trials, both across all four locations (response-congruent *M* = 579, response-incongruent *M* = 575), *F*(1,15) = 0.03, *p* > 0.25, and for the center two locations only (response-congruent *M* = 555, response-incongruent *M* = 555), *F*(1,15) = 0.00, *p* > 0.25; all interaction *F*’*s* < 2.5. Thus, congruency effects in the *orthogonal* task were driven by the alignment of anchor points, not by associated response mappings from the *single-dimension* tasks.

    To determine whether these congruency effects were related to the direction of initial eye movements, we next examined whether RTs differed for trials in which first saccades were directed towards the task-relevant anchor point when it appeared at the same location as the task-irrelevant preferred feature (congruent) versus the task-irrelevant non-preferred feature (incongruent) over the center two locations. We ran a repeated-measures ANOVA on RTs with experimental group (1a/1b) as a between-subjects factor and congruency (congruent or incongruent) as a within-subjects factor. There was a significant main effect of congruency, with faster RTs when first saccades were directed towards congruent versus incongruent stimuli (congruent *M* = 529, incongruent *M* = 557), *F*(1,15) = 10.09, *p* < 0.01; interaction *F* < 0.5. Thus, participants benefited from having anchor points from both dimensions appear in the same location as early as their first attentional shift.

     Having established an anchor point congruency effect at the response and spatial attention stages, we next tested whether the effect was present even at the saccade initiation stage. We ran the same ANOVA described above on saccade latency times but did not observe a main effect of congruency (*M* congruent = 262 ms, *M* incongruent = 268 ms), *F*(1,15) = 0.61, *p*  > 0.25. However, there was a trend for an interaction between experimental group and congruency, *F*(1,15) = 3.79, *p* = 0.07. Post-hoc tests revealed that saccade latency times were significantly faster for congruent (*M* = 258 ms) than incongruent targets (*M* = 279 ms) for the size-relevant task, *t*(8) = 3.42, *p* < 0.01, but not for the contrast-relevant task (*M* congruent = 265 ms, *M* incongruent = 240 ms), *t*(7) = 0.66, *p* > 0.25. In other words, task-irrelevant variations in contrast influenced saccade initiations during size-based judgments, but task-irrelevant variations in size did not influence saccade initiations during contrast-based judgments to the same extent.
