# Supplementary material for: Visual routines are associated with specific graph interpretations
Source: Cogn Res Princ Implic. 2017 Mar 20;2:20. doi: 10.1186/s41235-017-0059-2 (PMC5357663; doi:10.1186/s41235-017-0059-2)
Supplement: Supplementary file 2 — Visualizations of first saccades for four representative participants for each single-dimension task and for the orthogonal task. For the orthogonal task, size was task-relevant for Participants 1 and 2, and contrast was task-relevant for Participants 3 and 4. Examples of incongruent trials (anchor points appearing on different bars) are shown for the orthogonal task (e.g., Participant 1 = tall/light bar; Participant 2 = short/dark bar; Participant 3 = tall/dark bar; Participant 4 = short/light bar). (PDF 265 kb) [file 41235_2017_59_MOESM2_ESM.pdf]

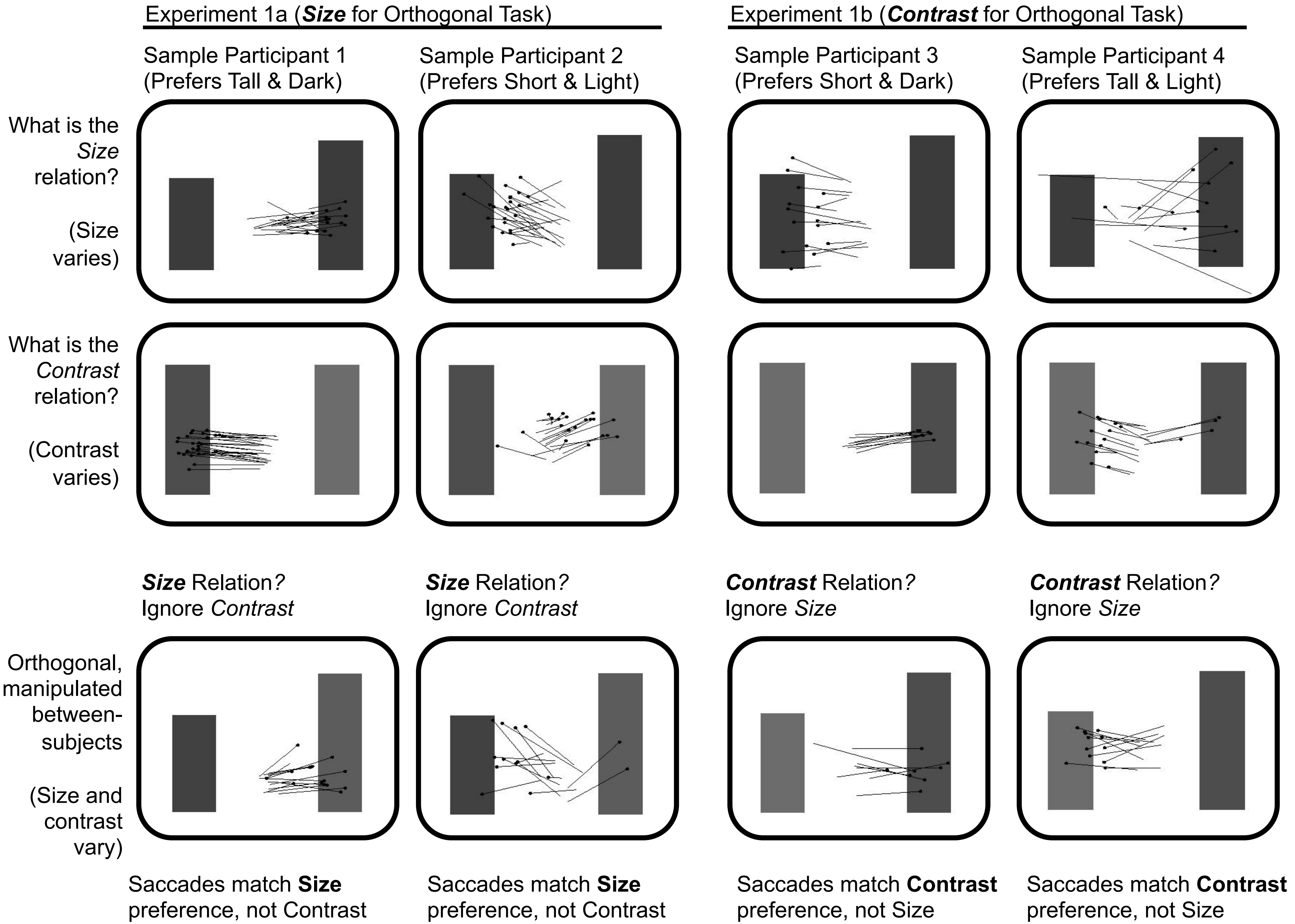

Experiment 1a (Size for Orthogonal Task)
Experiment 1b (Contrast for Orthogonal Task)
Sample Participant 1 (Prefers Tall & Dark)
Sample Participant 2 (Prefers Short & Light)
Sample Participant 3 (Prefers Short & Dark)
Sample Participant 4 (Prefers Tall & Light)
What is the Size relation?
(Size varies)
What is the Contrast relation?
(Contrast varies)
Size Relation? Ignore Contrast
Size Relation? Ignore Contrast
Contrast Relation? Ignore Size
Contrast Relation? Ignore Size
Orthogonal, manipulated between-subjects
(Size and contrast vary)
Saccades match Size preference, not Contrast
Saccades match Size preference, not Contrast
Saccades match Contrast preference, not Size
Saccades match Contrast preference, not Size
